# Personality and addictive behaviours in early Parkinson's disease and REM sleep behaviour disorder

**DOI:** 10.1016/j.parkreldis.2017.01.017

**Published:** 2017-04

**Authors:** Fahd Baig, Michael A. Lawton, Michal Rolinski, Claudio Ruffmann, Johannes C. Klein, Kannan Nithi, David Okai, Yoav Ben-Shlomo, Michele T.M. Hu

**Affiliations:** a Oxford Parkinson's Disease Centre, University of Oxford, Oxford, UK; b Nuffield Department of Clinical Neurosciences, University of Oxford, Oxford, UK; c School of Social and Community Medicine, University of Bristol, Bristol, UK; d Department of Neurology, Northampton General Hospital NHS Trust, Northampton, UK; e Psychological Medicine Service, Oxford University Hospitals NHS Trust, Oxford, UK

**Keywords:** Parkinson's disease, Personality, Addiction, REM sleep behaviour disorder, Smoking

## Abstract

**Introduction:**

Changes in personality have been described in Parkinson's disease (PD), with suggestion that those with established disease tend to be risk averse with a disinclination for addictive behaviour. However, little is known about the earliest and prodromal stages. Personality and its relationship with addictive behaviours can help answer important questions about the mechanisms underlying PD and addiction.

**Methods:**

941 population-ascertained PD subjects within 3.5 years of diagnosis, 128 patients with rapid eye movement sleep behaviour disorder (RBD) and 292 control subjects were fully characterised for motor symptoms, non-motor symptoms and across the following 5 personality domains: 1) neuroticism 2) extraversion 3) conscientiousness 4) agreeableness 5) openness using the Big Five Inventory.

**Results:**

Patients with early PD were more neurotic (p < 0.001), less extraverted (p < 0.001) and less open than controls (p < 0.001). RBD subjects showed the same pattern of being more neurotic (p < 0.001), less extraverted (p = 0.03) and less open (p < 0.001). PD patients had smoked less (p = 0.02) and drunk less alcohol (p = 0.03) than controls, but caffeine beverage consumption was similar. Being more extraverted (p < 0.001), more open (p < 0.001), and less neurotic (p < 0.001) predicted higher alcohol use, while being more extravert (p = 0.007) and less agreeable (p < 0.001) was associated with smoking more.

**Conclusions:**

A similar pattern of personality changes is seen in PD and RBD compared to a control population. Personality characteristics were associated with addictive behaviours, suggestive of a common link, but the lower rates of addictive behaviours before and after the onset of motor symptoms in PD persisted after accounting for personality.

## Highlights

• A similar pattern of personality change is seen in PD and RBD compared to controls.
• The similar pattern found suggests these personality changes occur before motor symptoms.
• Extraversion, linked with reward sensitivity, is associated with smoking and alcohol.
• Lower addictive behaviours before and after motor symptoms are not explained by personality alone.
• This suggests that inherent factors other than simple dopamine dysfunction drive these differences.

## 1 Introduction

While still controversial, a pre-morbid personality has been associated with Parkinson's disease (PD) since 1913 [1]. Assessment of personality varies depending on the model used, however some report that patients with established PD have a profile of less novelty seeking and more harm avoidance [2]. Other features relate to over controlled personality traits with introversion, mental rigidity, tenseness, social alertness and cautiousness [3]. It has been argued that reduced striatal dopaminergic signalling may cause these personality traits. There is also evidence that PD patients engage in less addictive behaviours than the general population, such as smoking, alcohol and caffeine use, which may or may not be secondary to these personality differences [4], [5]. Conversely, a significant proportion of patients can develop impulsive-compulsive behaviours (ICB) when treated with dopamine agonists [6]. In the general population, novelty seeking behaviour is associated with impulsive/addictive syndromes, linked to an exaggerated dopaminergic response to novel or rewarding stimuli [7]. These observations strengthen the view of dopamine as a central factor in both personality traits and reward or novelty based behaviour.

There have been few well-designed prospective studies investigating personality in this earliest stage of the disease process [2] or in the prodromal phase. The latter can be investigated with a cohort of patients with REM sleep behaviour disorder (RBD) because of their high risk of future conversion to PD and evidence, even at this stage, of a functional change in dopamine circuitry [8]. We have examined the following questions using the Oxford Discovery study comprising a large well-phenotyped PD cohort, RBD patients and population controls. (1) Does the personality of PD patients differ from controls in the early and pre-motor stages? (2) Can one explain differing patterns in addiction prone behaviours, such as smoking, caffeine and alcohol consumption in PD, by different personality profiles?

## 2 Materials and methods

### 2.1 Participants

Full details of the protocol have been described elsewhere [9]. In brief, PD patients diagnosed within 3.5 years were recruited between September 2010 and February 2014. Cases were eligible for inclusion if they met the UK PD Brain Bank criteria for diagnosis [10] irrespective of their age at PD onset, family history or cognitive status, while atypical cases were excluded. Cases diagnosed with dementia within one year of diagnosis were excluded as cases of Lewy body dementia.

The control population were clinically assessed to ensure they did not have PD or a first-degree relative with PD. The ‘RBD’ group comprised of participants with a diagnosis made by clinical assessment and polysomnography according to standard International Classification of Sleep Disorders-II criteria [11].

### 2.2 Clinical assessment

Personality was assessed using the Big Five Inventory (BFI-44). This is a self-rated questionnaire that uses a 5-point scale to rate 44 short phrases of character to evaluate the five factor model of personality: extraversion; neuroticism; agreeableness; openness; and conscientiousness (see supplemental tables 1 and 2) [12].

The addictive behaviours assessed were smoking, alcohol and caffeine use, each scored using the Mini Environmental Risk Questionnaire for PD. Current and past use for each was assessed, with consumption before diagnosis used for pre-morbid assessment in the PD group. A broad range of non-motor symptoms (NMS) were assessed, details of the tests and thresholds for positive symptoms are shown in supplemental table 3.

Motor function was assessed using MDS-UPDRS III and Hoehn and Yahr staging. PD patients were classified into three motor phenotypes (postural instability and gait difficulty (PIGD), tremor dominant (TD) and indeterminate) based on their MDS-UPDRS motor score [13].

We also collected data on socioeconomic status (type of accommodation and vehicle ownership) and education level (using years in formal education) to adjust for the potential confounding effect of socio-economic position on addiction prone behaviours that are socially patterned.

### 2.3 Statistical analysis

Continuous demographic variables were compared using ANOVA or Kruskall-Wallis tests (if distribution was not Gaussian). The chi-squared test was used for categorical data. Missing data was excluded from the analysis.

We initially tested whether there were differences in personality (outcome) by our three exposure groups (PD, RBD, controls). Each personality domain was categorised into quintiles and ordinal logistic regression was used to calculate the odds ratio (OR) for a unit change in the outcome as the assumptions required for linear regression were not valid. The Wald test of parallel lines assumptions was used to test the model was appropriate. We used multivariable models in our comparison between groups, incrementally adjusting for age, gender, affective disorders (anxiety and depression) and cognition. For comparison of PD subtypes, our regression models also included disease duration, motor severity and levodopa equivalent daily dose (LEDD).

We then tested whether disease status predicted our three addiction prone behaviours: smoking, alcohol and caffeine beverage consumption, which we had categorised into ordinal variables (see supplemental table 3). Our initial models adjusted for age, gender, socioeconomic status and educational level. We then adjusted for each of the 5 personality factors (the log of the total individual score was used due to non-normality) to see if this attenuated the associations. Finally to look at how personality predicted addiction prone behaviours, we pooled together all subjects (regardless of disease status) and adjusted for age, gender, socio-economic position and educational level.

We used a threshold of 0.05 as a level of statistical significance, but due to multiple testing, p-values between 0.05 and 0.001 should be interpreted with caution as they may reflect a type I error.

## 3 Results

**Fig. 1:**
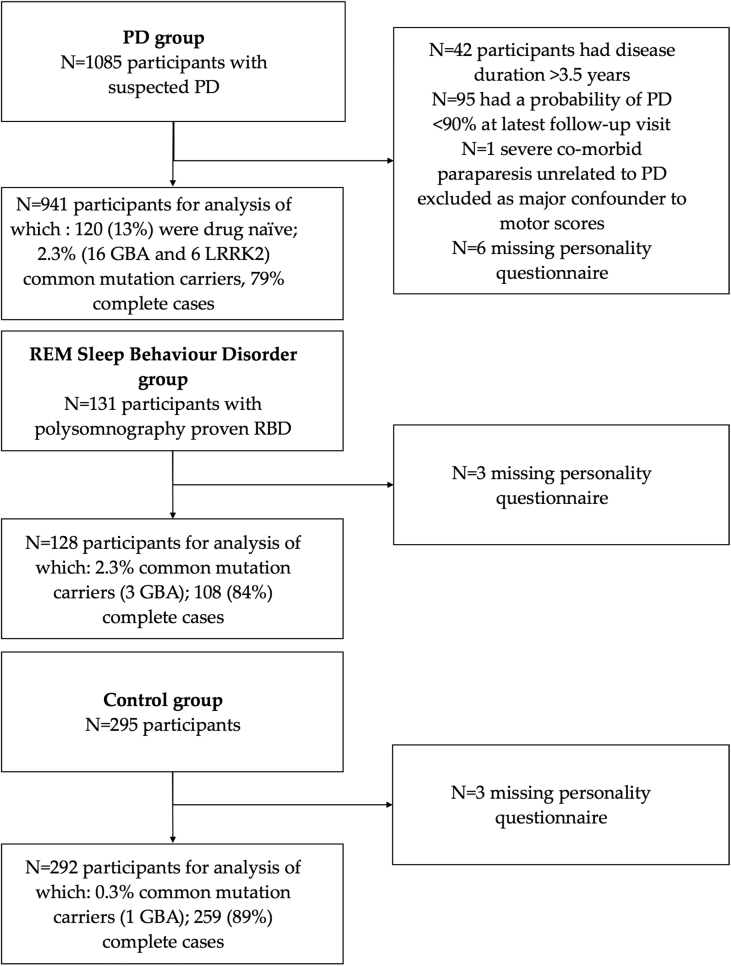
Flow chart of participant inclusion and exclusion.

Baseline data from 1361 participants (941 PD, 128 RBD and 292 controls) were included (Fig. 1). Basic demographics are shown in Table 1. Missing data in each variable was less than 3% except for the BDI (5.2%) and the QUIP-S (4.7%) leaving us with 1112 (81.7%) complete cases.

### 3.1 Comparison of personality in the early PD, RBD and control groups

The PD group was older than the control (p < 0.001) and the RBD groups (p = 0.01), which had similar age. There were substantially fewer women in the RBD group (13.3%) compared to both controls (51.0%) and PD group (35.2%) (both p values < 0.001) and the PD group also had fewer women than the controls. The control group were wealthier and more educated than both the PD and RBD groups (all p-values < 0.05). The PD group owned more of their own accommodation (p = 0.007) and had more bedrooms than the RBD group (p = 0.008).

Neuroticism and extraversion showed the biggest absolute differences between the control group and the others (see supplemental Fig. 1). Adjusting for age and gender, PD cases were more neurotic (OR 2.03, 95% CI 1.59, 2.58, p < 0.001), less extraverted (OR 0.53, 95% CI 0.42, 0.68, p < 0.001) and less open than the control group (OR 0.55, 95% CI 0.44, 0.70, p < 0.001) (see Table 2). The same pattern was seen when comparing the RBD group with controls, who were more neurotic (OR 3.07, 95% CI, 2.09, 4.52, p < 0.001), less extraverted (OR 0.65, 95% CI 0.44, 0.95, p = 0.03) and less open (OR 0.49, 95% CI 0.34, 0.72, p < 0.001). The addition of mood (depression and anxiety) and cognition as additional co-variates in the regression model had little effect on most of the results, except for neuroticism, which showed moderate attenuation with the addition of mood (OR for PD cases versus controls went from 2.03 to 1.49). Again the same pattern was seen in the RBD group following this adjustment, so they remained more neurotic and less open, but there was moderate attenuation for extraversion after including mood in the model (OR for RBD cases versus controls went from 0.65 to 0.85).

PD patients with the PIGD phenotype were less extraverted than the tremor dominant phenotype (OR 0.66, 95% CI 0.51, 0.87, p = 0.003) and more neurotic (OR 1.47, 95% CI 1.21,1.92, p = 0.005) but were otherwise similar (see supplementary Table 4). PD patients with RBD were more neurotic (OR 2.02, 95% CI 1.59, 2.58, p < 0.001), which was consistent with the other patterns, but were also less agreeable (OR 0.71, 95% CI 0.56, 0.90), p = 0.005) and less conscientious (OR 0.69, 95% CI 0.54,0.88, p = 0.002). Treated PD patients (not on dopamine replacement therapy) were less open than untreated PD patients (OR 0.64, 95% CI 0.44, 0.92, p = 0.02) but were otherwise similar (see supplementary Table 5). Differences between untreated PD patients and controls were attenuated compared to the whole PD group comparison, particularly the differences in openness were no longer evident (OR 0.81, 95% CI 0.55, 1.20, p = 0.30).

We undertook a number of sensitivity analyses by excluding younger (aged <50 years) or demented (MOCA <24) subjects in the comparison of personality between the patient groups, which had little effect on the results.

### 3.2 Smoking, alcohol and caffeine consumption

PD patients were less likely to have smoked regularly (at least 1 cigarette a day for 6 months) than controls (OR 0.72, 95% CI 0.54,0.96, p = 0.03). However, the difference was greater following diagnosis, with a greater proportion of PD patients having given up smoking (OR went from 0.72 to 0.44). Looking at total cigarette consumption (pack years smoked) (see Table 3), this showed the same pattern of PD patients smoking less than controls (OR 0.71, 95% CI 0.54, 0.93, p = 0.02). However, there was modest evidence that RBD patients possibly smoked more than controls (OR 1.55, 95% CI 1.01, 2.37, p = 0.05). Adjusting for personality in the model made little difference to the results except for alcohol consumption, which showed modest attenuation for PD versus controls (OR 0.70–0.79). PD patients with RBD symptoms may have had a modestly higher rate of smoking than those without (OR 1.33, 95% CI 0.99, 1.79, p = 0.06), but this difference was not statistically significant.

Personality traits were associated with addictive behaviours (see supplementary table 6). Being more extravert (OR 1.74, 95% CI 1.16, 2.60, p = 0.007) and less agreeable (OR 0.23, 95% CI 0.13, 0.56, p < 0.001) was associated with higher smoking consumption. Higher scores in extraversion (OR 2.14, 95% CI 1.44, 3.17), p < 0.001) and openness (OR 2.98, 95% CI 1.75, 5.06), p < 0.001), as well as lower neuroticism scores (OR 0.55, 95% CI 0.40, 0.76), p < 0.001), predicted higher alcohol consumption. These associations remained despite adjusting for mood and cognition, although the affect of neuroticism was less pronounced.

## 4 Discussion

### 4.1 Personality in PD and prodromal PD

Our findings support and build on some of the previous studies which suggest a ‘Parkinsonian personality’ is associated with being more introverted and neurotic. The presence of these personality differences in RBD and in the early stages of PD infers that these changes start before the onset of motor symptoms. Most previous studies have used personality assessments based on Cloninger's psychobiological model [14]. They demonstrated that PD patients had less novelty seeking and more harm avoidance characteristics than control populations [2], [15]. Using the five factor model, PD patients were shown to be more neurotic and less extraverted than controls if the patient was depressed [16]. Two other studies have shown less extraversion and conscientiousness in the PD group [17] or no differences in personality [18]. However these studies were small and potentially underpowered to detect differences. A major strength of this study is that the personality differences largely persisted despite adjusting for mood and cognition, which can affect the assessment of personality traits [15].

Sieurin et al. recently published the results of a prospective twin study which found neuroticism and introversion to be more common in subjects who converted to PD [19], in support of a prodromal PD personality. Postuma et al. found that RBD patients scored higher on harm avoidance than controls using the Tridimensional Personality Questionnaire [20]. Importantly these personality differences did not predict conversion to PD, implying that they remained relatively stable through the prodromal stage to conversion [21]. However Sasai et al. were unable to replicate these findings in an independent cohort using a five-factor model (NEO-PIR), although the study may have been too small to detect a difference [22]. This study adds considerable evidence to the concept of a prodromal personality profile, with the differences detected reflecting the same pattern of differences as the early PD group.

Proposed subtypes of PD may have different disease mechanisms, with the prevalence and severity of NMS being key distinguishing features. The same pattern of differences seen between PD and controls was again reflected in the differences between the PIGD and TD groups, consistent with a more severe NMS disease burden and more aggressive disease process. That PD patients with RBD were more neurotic than those without may also be a reflection of this, consistent with previous evidence that these patients have different phenotypic features [23], [24]. It remains unclear whether these differences are a direct result of the underlying pathophysiology or a consequence of disturbed sleep.

The attenuation of the differences, particularly in openness, between the untreated PD participants and controls, may be driven by these patients having a less aggressive disease process. As the study design recruited subjects with early Parkinson's rather than at the time of diagnosis, this result could just reflect a milder subtype of PD. The phenotypic differences between subtypes is more complex than primarily driven by dopamine dysfunction [25]. Thus while we have provided evidence of personality differences between subtypes, the similarities between treated and untreated PD cases may reflect that these affects are due to changes in alternative pathways.

### 4.2 Addictive behaviours in PD

There is a body of epidemiological evidence that suggests that PD is associated with lower rates of smoking [4], [26]. We had postulated that this inverse association with addictive behaviours may be a reflection of the personality traits associated with PD, driven by disruption of dopaminergic signalling. Analyses, when adjusted for differences in personality as a potential confounder, still found a robust difference in smoking rates between the PD group and controls. This suggests personality and certain reward-based activities are independent factors, consistent with the Swedish twin registry study (n = 197) [19] that also found a direct effect of neuroticism increasing the risk of PD using mediation analysis (i.e. conditioning out any indirect effect through a smoking mediator pathway).

PD patients were more likely to have given up smoking than controls, potentially a result of disease progression affecting reward sensitivity in this group. A similar finding was reported in early, untreated PD patients although we are unable to comment on the reasons for individuals quitting smoking [27].

Somewhat surprisingly, the RBD patients were more likely to have smoked than either the control or PD population, replicating the results of a previous large study of RBD patients [28]. The reasons for this are not clear and the trend of PD patients with RBD to smoke more than those without RBD replicates the finding of a previous study [29]. This may provide further support for PD heterogeneity with possibly different pathophysiological mechanisms or reflect behavioural changes due to the symptoms of the condition. A large population study using a screening questionnaire to diagnose probable RBD did not find this association with smoking, but this may be due to misclassification due to the assessment method [30].

Alcohol consumption was similar in the RBD group and controls, but less in the PD group. Personality accounted for more of the differences between the cases and controls than in the smoking comparison, but the overall affect was still small. Caffeine beverage consumption was similar across groups, possibly because the absolute caffeine value of different drinks was not assessed.

Extraversion was the only trait that predicted both higher smoking and alcohol consumption. Being more extravert is thought to represent an underlying sensitivity to reward, thus its association with addictive behaviour is intuitive. In addition to extraversion, openness was positively associated with higher alcohol use and neuroticism was inversely associated. This pattern of personality traits predicting more alcohol use is the inverse of the personality differences between cases and controls which suggests a common link between the mechanisms underlying personality differences and addictive behaviour in PD. Differences in how individual personality traits (particularly extraversion) are associated with smoking and alcohol may be linked by reward sensitivity. This is potentially driven by dysfunction of dopaminergic signalling in PD, which mediates reward sensitivity. Personality changes do not seem to fully explain these differences, potentially a result of these changes evolving due to more pathways than just dopamine dysfunction.

Major strengths of our study include: (i) The sizeable numbers of both RBD patients and a control population for direct comparison (ii) This is one of the largest studies published examining the personality type in the PD population (iii) The inclusion of patients with incident cognitive impairment is a strength, as their exclusion may bias the findings to milder PD subtypes. There are several study limitations that need to be considered. (i) The five-factor model of personality is widely accepted, however the ‘short form’ of assessment was used due to practical constraints. (ii) Whilst the attempt was made to adjust for demographic differences between groups, there may still be residual confounding differences. The Swedish twin study found much weaker associations between personality and PD when they did a within-twin pair analysis, suggesting familial confounding factors may partially generate the observed association in unrelated individuals [19]. (iii) Our observations may be susceptible to recall bias, although the results are consistent with the few prospective studies that have collected data on personality and addiction prone behaviours well before disease onset. (iv) It is uncertain which RBD patients will convert to PD and when. However, this would likely reduce rather than exaggerate any potential ‘prodromal’ characteristics of PD.

## 5 Conclusion

This study supports the concept of personality differences between PD and control subjects, even in the earliest stages of the motor phase of the disease. The similar pattern found in RBD patients (a surrogate for prodromal PD) is strongly suggestive of these personality changes occurring before motor symptom onset. Extraversion, which has been linked with reward sensitivity, is positively associated with smoking and alcohol consumption. The lower rates of addictive behaviours before and after the onset of motor symptoms in PD are not explained by the personality changes alone, at least as measured by the five factor model. However, the same personality characteristics that are affected in PD are associated with addictive behaviours, which is strongly suggestive of a common link.

## Ethics approval

Ethical approval for this study was granted by the Berkshire Ethics Committee, South Central, National Research Ethics Service (UK): reference number 10/H0505/71. All participating subjects provided informed written consent.

## Financial disclosure/conflict of interest

The authors have no conflicts of interest to declare. The OPDC Discovery cohort is funded by the Monument Trust Discovery Award from Parkinson's UK (grant number J-1403) and supported by the National Institute for Health Research (NIHR) Oxford Biomedical Research Centre based at Oxford University Hospitals NHS Trust and University of Oxford, and the Dementias and Neurodegenerative Diseases Research Network (DeNDRoN).

## Full financial disclosures of all authors for the past year

FB – Employed by the Oxford Parkinson's Disease Centre, UK.

ML – Employed by the School of Social and Community Medicine, UK.

MR – Funded by the British Research Council, UK.

CR - Employed by the Oxford Parkinson's Disease Centre, UK.

JK - Employed by the Oxford Parkinson's Disease Centre, UK.

KN – Employed by Northampton General Hospital, UK.

DO – Employed by Oxford University Hospitals Trust, UK.

YBS - Advisory Boards: member of the Multiple Sclerosis -risk sharing scheme scientific advisory board. Employed by the University of Bristol. Royalties from books published by Oxford University Press and Wiley. Grants received from Parkinson's UK, Cancer Research UK, National Institute of Health Research, British Heart Foundation and Medical Research Council.

MH – Funded by OPDC Monument Discovery award, the Oxford Biomedical Research Centre and National Institute of Health Research Clinical Research Network.

## Author roles

1. Research Project: A. Conception, B. Organization, C. Execution; 2. Statistical Analysis: A. Design, B. Execution, C. Review and Critique; 3. Manuscript Preparation: A. Writing the First Draft, B. Review and Critique.

FB: 1A, 1C, 2A, 2B, 3A.

ML: 2A, 2B, 2C, 3B.

MR: 1C, 3B.

CR: 1C, 3B.

JK: 1C, 3B.

KN: 1C, 3B.

DO: 1A, 2C, 3B.

YBS: 1A, 1B, 2B, 2C, 3B.

MH: 1A, 1B, 1C, 2C, 3B.

## Competing interests

None.

## Figures and Tables

**Table 1:** Demographics of each subject group.

	PD (n = 941)	RBD (n = 128)	Controls (n = 292)
Age (mean, range (SD))	67.2, 32-90 (9.6)	65.0, 29-81 (8.9)	65.1, 28–88 (10.0)
Gender (female n (%))	332 (35.2)	17 (13.3)	148 (51.0)
Ethnicity (non-white n (%))	20 (2.1)	3 (2.3)	6 (2.1)
First-degree relatives with PD (n (%))	147 (15.7)	8 (6.3)	0
Age of PD motor symptom onset (mean, range (SD))	64.3, 20-87 (9.8)	n/a	n/a
Disease duration from PD diagnosis in years (mean, range (SD))	1.3, 0.01–3.5 (0.9)	n/a	n/a
MDS-UPDRS III (mean (SD))	26.4 (10.8)	4.3 (4.3)	1.7 (2.7)
Hoehn and Yahr Stage (n (%))			
1	215 (23.0)	n/a	n/a
2	658 (70.2)	n/a	n/a
3	64 (6.8)	n/a	n/a
Untreated PD (n (%))	120 (12.8)	n/a	n/a
Levodopa equivalent daily dosage (treated patients only) (mean (SD))	323 (196)	n/a	n/a
Treated participants were on the following medications (n (%))^a^:			
Levodopa	519 (63.3)	n/a	n/a
Dopamine agonist	280 (34.2)		
MAOB-I	229 (27.9)		
Cognition (Montreal Cognitive Assessment) (mean, median (interquartile range))	25.0, 25 (23–27)	25.3, 26 (24–27)	26.7 (25–29)
Depression (Beck's Depression Inventory-II) (mean, median (interquartile range))	8.8, 8 (4–12)	9.8, 6 (2–15)	4.8, 4 (1–7)
Depression (Beck's Depression Inventory-II) (positive screen (n (%))	157 (17.8)	34 (27.6)	18 (6.4)
Anxiety (Leeds Anxiety and Depression Scale) (mean, median (interquartile range))	3.4, 3 (1–5)	4.1, 3.5 (1–6)	2.1, 2 (0–3)
Anxiety (Leeds Anxiety and Depression Scale) positive screen (n (%))	159 (17.2)	27 (21.4)	17 (5.9)
Impulse Control Behaviours (QUIP-S)^b^ positive screen (n (%))	195 (22.0)	40 (32.0)	61 (21.5)
RBD^c^ (RBD Sleep Questionnaire) (mean, median (interquartile range))	4.8, 4 (2–7)	10.1, 10 (9–12)	2.7, 2 (1–4)
Daytime somnolence (Epworth Sleepiness Scale) (mean, median (interquartile range))	7.6, 7 (4–10)	7.3, 6.5 (4–10)	5.7, 5 (3–8)
Ever smoked (n (%))	382 (40.8)	82 (64.1)	127 (43.5)
Current smokers (n (%))	23 (2.5)	10 (7.8)	13 (4.5)
Smoking history in pack years (smokers only) (mean, median (interquartile range)	15.8, 10 (3.75–21)	27.0, 15 (6–40)	14.5, 10 (3.5–20)
Prior alcohol use in units per week (mean, median (interquartile range)	10.5, 6 (1–14)	16.2, 9.5 (2–24)	11.4, 8 (2–18)
Current alcohol use in units per week (mean, median (interquartile range)	7.9, 4 (0–10)	8.9, 4.5 (0–12)	9.5, 6 (1–14)
Prior caffeine use in total beverages per day (mean, median (interquartile range)	4.8, 5 (3–6)	5.3, 5 (3–6)	4.7, 5 (3–6)
Current caffeine use in total beverages per day (mean, median (interquartile range)	4.1, 4 (2–6)	3.9, 4 (3–5)	4.1, 4 (3–6)
Number of vascular risk factors^d^(n (%))			
0	434 (46.3)	55 (43.3)	157 (53.8)
1	260 (27.7)	25 (19.7)	70 (24.0)
>2	244 (26.0)	47 (37.0)	65 (22.3)
Big Five Inventory:			
Extraversion (mean (SD))	24.3 (6.7)	25.0 (6.5)	27.1 (6.7)
Neuroticism (mean (SD))	22.2 (6.6)	24.1 (7.3)	19.8 (6.7)
Agreeableness (mean (SD))	36.8 (5.1)	34.8 (5.6)	36.8 (4.9)
Openness (mean (SD))	34.8 (7.1)	34.5 (6.6)	37.2 (6.9)
Conscientiousness (mean (SD))	35.8 (5.8)	35.0 (6.3)	36.4 (5.8)
Social Background:			
Accommodation owned, (n, %)	859 (91.7)	108 (84.4)	277 (95.2)
More than 3 Bedrooms in accommodation, (n, %)	407 (44.5)	40 (32.0)	151 (52.4)
More than 1 vehicle owned, (n, %)	458 (50.3)	53 (43.4)	158 (55.1)
Number of years in formal education (mean, median, interquartile range)	14.0, 14 (11–16)	13.8, 13 (11–16)	15.1, 16 (12–17)

a Percentages relate to the number on each drug, some patients are on more than one class of drug.

b Questionnaire for Impulsive-Compulsive Disorders in Parkinson's Disease.

c Rapid Eye Movement Sleep Behaviour Disorder.

d Includes angina, heart failure, stroke or TIA, heart attack, diabetes, hypercholesterolaemia and hypertension.

**Table 2:** Comparison of personality type between subject groups.

Co-variates included in model	PD vs Controls (OR (95% CI); p value)			RBD vs Controls (OR (95% CI); p value)		
	Age and gender	Age, gender and mood	Age, gender, mood and cognition	Age and gender	Age, gender and mood	Age, gender, mood and cognition
Extraversion	0.53 (0.42–0.68), p < 0.001	0.60 (0.47–0.77), p < 0.001	0.61 (0.47–0.78), p < 0.001	0.65 (0.44–0.95), p = 0.03	0.85 (0.57–1.26), p = 0.42	0.87 (0.58–1.30), p = 0.50
Neuroticism	2.03 (1.59–2.58), p < 0.001	1.49 (1.16–1.92), p = 0.002	1.49 (1.16–1.92), p = 0.002	3.07 (2.09–4.52), p < 0.001	1.91 (1.27–2.86), p = 0.002	1.93 (1.29–2.90), p = 0.001
Agreeableness	1.12 (0.89–1.42), p = 0.33	1.25 (0.98–1.60), p = 0.07	1.23 (0.97–1.59), p = 0.09	0.74 (0.51–1.08), p = 0.12	0.91 (0.61–1.35), p = 0.64	0.93 (0.62–1.38), p = 0.70
Openness	0.55 (0.44–0.70), p < 0.001	0.54 (0.43–0.70), P < 0.001	0.57 (0.44–0.73), p < 0.001	0.49 (0.34–0.72), p < 0.001	0.56 (0.38–0.83), p = 0.004	0.57 (0.38–0.84), p = 0.005
Conscientiousness	0.86 (0.68–1.08), p = 0.20	0.99 (0.77–1.27), p = 0.93	1.03 (0.80–1.32), p = 0.80	0.77 (0.52–1.12), p = 0.18	0.99 (0.67–1.47), p = 0.96	0.99 (0.67–1.48), p = 0.97

The total score for each personality trait was divided into quintiles with ordinal logistic regression then used to calculate odds ratios. The co-variates for each regression model is listed.

**Table 3:** Comparison of smoking, alcohol and caffeine between groups.

	Pre-morbid/Past Use			Current Use	
	Smoking	Alcohol	Caffeine	Alcohol	Caffeine
PD vs Controls (OR (95% CI); p value)	0.71 (0.54–0.93), p = 0.02	0.70 (0.53–0.91), p = 0.009	1.00 (0.78–1.29), p = 0.98	0.59 (0.45–0.77), p < 0.001	0.92 (0.71–1.18), p = 0.50
PD vs Controls (OR (95% CI); p value) [adjusting for personality]	0.73 (0.55–0.97), p = 0.03	0.79 (0.60–1.05), p = 0.11	0.93 (0.71–1.21), p = 0.58	0.66 (0.50–0.87), p = 0.004	0.85 (0.65–1.11), p = 0.24

RBD vs Controls (OR (95% CI); p value)	1.55 (1.01–2.37), p = 0.05	0.94 (0.60–1.47), p = 0.78	1.16 (0.78–1.75), p = 0.48	0.60 (0.39–0.93), p = 0.02	0.78 (0.52–1.18), p = 0.24
RBD vs Controls (OR (95% CI); p value) [adjusting for personality]	1.62 (1.04–2.53), p = 0.03	0.97 (0.61–1.54), p = 0.89	1.15 (0.75–1.77), p = 0.52	0.64 (0.40–1.01), p = 0.06	0.78 (0.51–1.18), p = 0.24

The number of pack years smoked, weekly alcohol intake (current and past) and daily caffeine intake (current and past) were used for analysis. There were not enough current smokers to model current smoking behaviours between groups.

Ordered logistic regression was used to calculate odds ratios, adjusted for age, gender and socio-economic position to compare the patient groups using pre-morbid or current consumption levels. Each of the five factors were included in the model as co-variates in addition to compare the effects of personality on addictive behaviours between the groups.
