# Supplementary material for: Personality and addictive behaviours in early Parkinson's disease and REM sleep behaviour disorder
Source: Parkinsonism Relat Disord. 2017 Apr;37:72–8. doi: 10.1016/j.parkreldis.2017.01.017 (PMC5380654; doi:10.1016/j.parkreldis.2017.01.017)
Supplement: Supplementary material [file mmc1.docx]

# SUPPLEMENTARY MATERIAL – Personality in Early Parkinson’s Disease.

### SUPPLEMENTAL TABLE 1 – The Big Five Inventory [1]

**Here are a number of characteristics that may or may not apply to you. For example, do you agree that you are someone who likes to spend time with others? Please tick ONLY ONE BOX next to each statement to indicate the extent to which you agree or disagree with that statement.**

|  |  | Disagree  strongly | | Disagree  a little | | Neither agree nor  disagree | | Agree  a little | | Agree  strongly | |
| --- | --- | --- | --- | --- | --- | --- | --- | --- | --- | --- | --- |
| **1.** | Is talkative | □_1_ | | □_2_ | | □_3_ | | □_4_ | | □_5_ | |
| **2.** | Tends to find fault with others | □_1_ | | □_2_ | | □_3_ | | □_4_ | | □_5_ | |
| **3.** | Does a thorough job | □_1_ | | □_2_ | | □_3_ | | □_4_ | | □_5_ | |
| **4.** | Is depressed, blue | □_1_ | | □_2_ | | □_3_ | | □_4_ | | □_5_ | |
| **5.** | Is original, comes up with new ideas | □_1_ | | □_2_ | | □_3_ | | □_4_ | | □_5_ | |
| **6.** | Is reserved | □_1_ | | □_2_ | | □_3_ | | □_4_ | | □_5_ | |
| **7.** | Is helpful and unselfish with others | □_1_ | | □_2_ | | □_3_ | | □_4_ | | □_5_ | |
| **8.** | Can be somewhat careless | □_1_ | | □_2_ | | □_3_ | | □_4_ | | □_5_ | |
| **9.** | Is relaxed, handles stress well | □_1_ | | □_2_ | | □_3_ | | □_4_ | | □_5_ | |
| **10.** | Is curious about many different things | □_1_ | | □_2_ | | □_3_ | | □_4_ | | □_5_ | |
| **11.** | Is full of energy | □_1_ | | □_2_ | | □_3_ | | □_4_ | | □_5_ | |
| **12.** | Starts quarrels with others | □_1_ | | □_2_ | | □_3_ | | □_4_ | | □_5_ | |
| **13.** | Is a reliable worker | □_1_ | | □_2_ | | □_3_ | | □_4_ | | □_5_ | |
| **14.** | Can be tense | □_1_ | | □_2_ | | □_3_ | | □_4_ | | □_5_ | |
| **15.** | Is ingenious, a deep thinker | □_1_ | | □_2_ | | □_3_ | | □_4_ | | □_5_ | |
| **16.** | Generates a lot of enthusiasm | □_1_ | | □_2_ | | □_3_ | | □_4_ | | □_5_ | |
| **17.** | Has a forgiving nature | □_1_ | | □_2_ | | □_3_ | | □_4_ | | □_5_ | |
| **18.** | Tends to be disorganized | □_1_ | | □_2_ | | □_3_ | | □_4_ | | □_5_ | |
| **19.** | Worries a lot | □_1_ | | □_2_ | | □_3_ | | □_4_ | | □_5_ | |
| **20.** | Has an active imagination | □_1_ | | □_2_ | | □_3_ | | □_4_ | | □_5_ | |
| **21.** | Tends to be quiet | □_1_ | | □_2_ | | □_3_ | | □_4_ | | □_5_ | |
| **22.** | Is generally trusting | | □_1_ | | □_2_ | | □_3_ | | □_4_ | | □_5_ |
| **23.** | Tends to be lazy | | □_1_ | | □_2_ | | □_3_ | | □_4_ | | □_5_ |
| **24.** | Is emotionally stable, not easily upset | | □_1_ | | □_2_ | | □_3_ | | □_4_ | | □_5_ |
| **25.** | Is inventive | | □_1_ | | □_2_ | | □_3_ | | □_4_ | | □_5_ |
| **26.** | Has an assertive personality | | □_1_ | | □_2_ | | □_3_ | | □_4_ | | □_5_ |
| **27.** | Can be cold and aloof | | □_1_ | | □_2_ | | □_3_ | | □_4_ | | □_5_ |
| **28.** | Perseveres until the task is finished | | □_1_ | | □_2_ | | □_3_ | | □_4_ | | □_5_ |
| **29.** | Can be moody | | □_1_ | | □_2_ | | □_3_ | | □_4_ | | □_5_ |
| **30.** | Values artistic, aesthetic experiences | | □_1_ | | □_2_ | | □_3_ | | □_4_ | | □_5_ |
| **31.** | Is sometimes shy, inhibited | | □_1_ | | □_2_ | | □_3_ | | □_4_ | | □_5_ |
| **32.** | Is considerate and kind to almost everyone | | □_1_ | | □_2_ | | □_3_ | | □_4_ | | □_5_ |
| **33.** | Does things efficiently | | □_1_ | | □_2_ | | □_3_ | | □_4_ | | □_5_ |
| **34.** | Remains calm in tense situations | | □_1_ | | □_2_ | | □_3_ | | □_4_ | | □_5_ |
| **35.** | Prefers work that is routine | | □_1_ | | □_2_ | | □_3_ | | □_4_ | | □_5_ |
| **36.** | Is outgoing, sociable | | □_1_ | | □_2_ | | □_3_ | | □_4_ | | □_5_ |
| **37.** | Is sometimes rude to others | | □_1_ | | □_2_ | | □_3_ | | □_4_ | | □_5_ |
| **38.** | Makes plans and follows through with them | | □_1_ | | □_2_ | | □_3_ | | □_4_ | | □_5_ |
| **39.** | Gets nervous easily | | □_1_ | | □_2_ | | □_3_ | | □_4_ | | □_5_ |
| **40.** | Likes to reflect, play with ideas | | □_1_ | | □_2_ | | □_3_ | | □_4_ | | □_5_ |
| **41.** | Has few artistic interests | | □_1_ | | □_2_ | | □_3_ | | □_4_ | | □_5_ |
| **42.** | Likes to cooperate with others | | □_1_ | | □_2_ | | □_3_ | | □_4_ | | □_5_ |
| **43.** | Is easily distracted | | □_1_ | | □_2_ | | □_3_ | | □_4_ | | □_5_ |
| **44.** | Is sophisticated in art, music, or literature | | □_1_ | | □_2_ | | □_3_ | | □_4_ | | □_5_ |

**BFI scale scoring (“R” denotes reverse-scored items):**

Extraversion: 1, 6R, 11, 16, 21R, 26, 31R, 36

Agreeableness: 2R, 7, 12R, 17, 22, 27R, 32, 37R, 42

Conscientiousness: 3, 8R, 13, 18R, 23R, 28, 33, 38, 43R

Neuroticism: 4, 9R, 14, 19, 24R, 29, 34R, 39

Openness: 5, 10, 15, 20, 25, 30, 35R, 40, 41R, 44

**SUPPLEMENTAL TABLE 2 – Big five personality dimensions [2]**

| **Big Five Dimensions** | **Facet (and correlated trait adjective)** |
| --- | --- |
| Extraversion vs. introversion | Gregariousness (sociable)  Assertiveness (forceful)  Activity (energetic)  Excitement-seeking (adventurous)  Positive emotions (enthusiastic)  Warmth (outgoing) |
| Agreeableness vs. antagonism | Trust (forgiving)  Straightforwardness (not demanding)  Altruism (warm)  Compliance (not stubborn)  Modesty (not show-off)  Tender-mindedness (sympathetic) |
| Conscientiousness vs. lack of direction | Competence (efficient)  Order (organized)  Dutifulness (not careless)  Achievement striving (thorough)  Self-discipline (not lazy)  Deliberation (not impulsive) |
| Neuroticism vs. emotional stability | Anxiety (tense)  Angry hostility (irritable)  Depression (not contented)  Self-consciousness (shy)  Impulsiveness (moody)  Vulnerability (not self-confident) |
| Openness vs. closedness to experience | Ideas (curious)  Fantasy (imaginative)  Aesthetics (artistic)  Actions (wide interests)  Feelings (excitable)  Values (unconventional) |

This table compares the Big Five dimensions personality used in this study with the facets of personality as described by the revised NEO personality inventory

### SUPPLEMENTAL TABLE 3 - Clinical metrics used for assessment of non-motor symptoms, motor symptoms, and health related quality of life.

|  | **Scale / Clinical assessment** |
| --- | --- |
| **NON-MOTOR SYMPTOMS (NMS)** |  |
| **Neuropsychiatric** |  |
| Depression and anxiety assessment | Leeds Scales for the Self Assessment of Anxiety and Depression: using a threshold of >7 for to score positive.[3]  Beck Depression Inventory II: using scores of 0-13 to denote minimal depression, and scores >14 were taken as positive for depression (including mild, moderate and severe categories).[4] |
| Cognition | Montreal Cognitive Assessment tool (MOCA): using screening cut-off scores of 22-23 to denote possible mild cognitive impairment (MCI) and <22 for possible dementia.[5] |
| Impulse control behaviours | The shortened Questionnaire for Impulsive-Compulsive Disorders in Parkinson’s Disease (QUIP-S): using a positive response in any category as a positive screen for impulse control behaviours.[6] |
| **Gastrointestinal** |  |
| Constipation symptoms | The constipation questionnaire as based on the Honolulu-Asia Ageing Study: <1 daily bowel movement or use of laxatives were deemed constipated.[7] The MDS-UPDRS part I question 1.11 was also used to compare constipation symptoms with laxative use. |
| **Sleep** |  |
| REM sleep behaviour disorder | Rapid Eye Movement Sleep Behaviour Disorder Screening Questionnaire (RBDSQ): using a threshold of ≥6 for a positive screen in the early PD and RBD, and ≥5 in controls.[8] |
| Daytime somnolence symptom | Epworth Sleepiness Scale (ESS): using a threshold of ≥10 as a positive screen.[9] |
| **Autonomic** |  |
| Orthostatic hypotension assessment | Blood pressure was measured after the patient lies flat for 3 minutes then again after standing upright for 2 minutes: a systolic drop of blood pressure (BP) of ≥20mmHg or a diastolic drop of ≥10mmHg were deemed positive.[10] |
| **Sensory** |  |
| Pain | EQ5D: Question 5: A 3 point scale of no, moderate or extreme pain or discomfort. Moderate or extreme pain were deemed positive.[11] |
| Hyposmia assessment | Sniffin sticks odour identification test dichotomised at 10th percentile according to normal values for age and sex:[12]  Males and females age 16-≤36: 11 or lower;  Males aged >36-≤55: 12 or lower;  females aged >36-≤55: 11 or lower;  Males and females age >55: 9 or lower. |
| **Total NMS Score** |  |
| Comparison between PD and control groups | The sum total of 9 NMS domains assessed was used: cognition; anxiety; depression; ICB; constipation; orthostatic hypotension; pain; hyposmia; daytime somnolence; and RBD.  Where a single item was missing from a case that symptom was imputed as negative and the case included in the total score. |
| **MOTOR SYMPTOMS** |  |
| UPDRS part III (motor symptoms score) | The MDS-UPDRS uses a scale of 0-4, each score relating to an absent, mild, moderate or severe motor symptom. The motor score part III examination includes assessments of rigidity, bradykinesia, tremor, gait and postural instability with other features.[13] |
| **QUALITY OF LIFE** |  |
| EQ-5D-3L | The 5 domains of mobility, self-care, usual activities, pain and mood each on a 3 point scale which is then converted into a summary index score for comparison.[11] The summary index was then stratified into quintiles for analysis. |
| **SUBSTANCE USE** |  |
| Mini Environmental Risk Questionnaire for PD Baseline (MERQ-PD B) | Assessment for exposure to commonly accepted risk factors for PD was used to assess current caffeine, alcohol and smoking consumption, while premorbid consumption was also assessed as prior to PD diagnosis or in the past if no diagnosis of PD.[14]  For total exposure total pack years of smoking were split into 3 categories of never smoked; 1-10 pack years history; and ≥10 pack years history.  The total units of alcohol consumed in a week was calculated and split into 3 categories: 0 units per week; 1-14 units per week; 15+ units per week (based on UK alcohol guidelines).  The number of caffeine beverages consumed in a day for each individual was calculated and split into 3 categories: 0-3 cups per day, 4-5 cups per day and 6 or more cups per day (to represent no or minimal consumption, moderate and heavier consumption). |
| **SOCIAL BACKGROUND** |  |
| Years in education | Total years in education including school and higher education whether full or part-time. For inclusion in analysis, this was stratified into quintiles due to non-normality. |
| Accommodation owned at diagnosis | Dichotomised to ‘accommodation rented’ scored as 0 versus ‘accommodation owned or have a mortgage’ scored as 1. |
| More than 3 Bedrooms in accommodation at diagnosis | Dichotomised to ‘≤3’ scored as 0 versus ‘>3’ scored as 1. |
| Vehicles owned at diagnosis | Dichotomised to ‘≤1’ scored as 0 versus ‘>1’ scored as 1. |

1. Soto CJ, John OP. Ten facet scales for the Big Five Inventory: Convergence with NEO PI-R facets, self-peer agreement, and discriminant validity. *Journal of Research in Personality* 2009;**43**(1):84-90.

2. John OP, Naumann LP, Soto CJ. Paradigm shift to the integrative big five trait taxonomy. *Handbook of personality: Theory and research* 2008;**3**:114-58.

3. Snaith RP, Bridge GW, Hamilton M. The Leeds scales for the self-assessment of anxiety and depression. *Br. J. Psychiatry* 1976;**128**:156-65.

4. Beck AT, Ward C, Mendelson M. Beck depression inventory (BDI). *Arch. Gen. Psychiatry* 1961;**4**(6):561-71.

5. Hu MT, Szewczyk-Krolikowski K, Tomlinson P, et al. Predictors of cognitive impairment in an early stage Parkinson's disease cohort. *Mov. Disord.* 2014

6. Weintraub D, Hoops S, Shea JA, et al. Validation of the questionnaire for impulsive-compulsive disorders in Parkinson's disease. *Mov. Disord.* 2009;**24**(10):1461-7.

7. Abbott RD, Ross GW, White LR, et al. Environmental, life-style, and physical precursors of clinical Parkinson's disease: recent findings from the Honolulu-Asia Aging Study. *J. Neurol.* 2003;**250 Suppl 3**:III30-9.

8. Nomura T, Inoue Y, Kagimura T, et al. Utility of the REM sleep behavior disorder screening questionnaire (RBDSQ) in Parkinson's disease patients. *Sleep Med.* 2011;**12**(7):711-3.

9. Johns MW. A new method for measuring daytime sleepiness: the Epworth sleepiness scale. *Sleep* 1991;**14**(6):540-5.

10. Freeman R, Wieling W, Axelrod FB, et al. Consensus statement on the definition of orthostatic hypotension, neurally mediated syncope and the postural tachycardia syndrome. *Auton. Neurosci.* 2011;**161**(1-2):46-8.

11. EuroQol G. EuroQol--a new facility for the measurement of health-related quality of life. *Health Policy* 1990;**16**(3):199-208.

12. Hummel T, Kobal G, Gudziol H, et al. Normative data for the "Sniffin' Sticks" including tests of odor identification, odor discrimination, and olfactory thresholds: an upgrade based on a group of more than 3,000 subjects. *Eur. Arch. Otorhinolaryngol.* 2007;**264**(3):237-43.

13. Goetz CG, Tilley BC, Shaftman SR, et al. Movement Disorder Society-Sponsored Revision of the Unified Parkinson's Disease Rating Scale (MDS-UPDRS): Scale Presentation and Clinimetric Testing Results. *Mov. Disord.* 2008;**23**(15):2129-70.

14. PD-DOC study / NIH common data elements: http://grants.nih.gov/grants/guide/rfa-files/RFA-NS-11-001.html. Date accessed 24/11/2015.

<http://www.commondataelements.ninds.nih.gov>

At the date this paper was written, URLs or links referenced herein were deemed to be useful supplementary material to this paper. Neither the author nor the journal warrants or assumes liability for the content or availability of URLs referenced in this paper.

### SUPPLEMENTAL TABLE 4 – Comparison of personality type between PD subtypes

| **Personality domain** | **PIGD vs TD**  **(OR(95% CI); p value)** | **PD+RBD vs PD-RBD (OR(95% CI); p value)** |
| --- | --- | --- |
| Extraversion | 0.66 (0.51-0.87), p=0.003 | 1.09 (0.86-1.39), p=0.48 |
| Neuroticism | 1.47 (1.21-1.92), p=0.005 | 2.02 (1.59-2.58), p<0.001 |
| Agreeableness | 0.78 (0.60-1.01), p=0.06 | 0.71 (0.56-0.90), p=0.005 |
| Openness | 0.73 (0.56-0.96), p=0.02 | 1.05 (0.83-1.34), P=0.67 |
| Conscientiousness | 0.90 (0.69-1.17), p=0.43 | 0.69 (0.54-0.88), p=0.002 |

Ordinal logistic regression was used to calculate odds ratios, adjusted for age, gender, disease duration from diagnosis, motor severity, and LEDD. The motor subtypes (postural instability gait disorder (PIGD) and tremor dominant (TD)) and PD participants with (PD+RBD) and without (PD-RBD) REM sleep behaviour disorder were compared

### SUPPLEMENTAL TABLE 5 – Comparison of PD patients not on dopaminergic therapy with treated PD patients and controls.

| **Personality domain** | **PD on treatment vs Untreated PD (OR(95% CI); p value)** | **Untreated PD vs Controls**  **(OR(95% CI); p value)** |
| --- | --- | --- |
| Extraversion | 0.79 (0.55-1.13), p=0.20 | 0.69 (0.47-1.02), p=0.06 |
| Neuroticism | 1.12 (0.77-1.62), p=0.54 | 1.84 (1.24-2.73), p=0.002 |
| Agreeableness | 0.96 (0.67-1.37), p=0.82 | 0.13 (0.77-1.66), p=0.54 |
| Openness | 0.64 (0.44-0.92), P=0.02 | 0.81 (0.55-1.20), p=0.30 |
| Conscientiousness | 0.82 (0.56-1.18), p=0.28 | 0.97 (0.66-1.43), p=0.88 |

Ordinal logistic regression was used to calculate odds ratios, adjusted for age and gender, with the additional co-variates disease duration from diagnosis and motor severity included for comparing treated and untreated PD patients.

## SUPPLEMENTAL TABLE 6 – The effect of personality on smoking, alcohol and caffeine use

|  | **All groups (OR(95% CI); p value)** | | | | | |
| --- | --- | --- | --- | --- | --- | --- |
| **Co-variates included in model** | **Age, gender and socio-economic position** | | | **Age, gender, mood, cognition and socio-economic position** | | |
|  | **Smoking** | **Alcohol** | **Caffeine** | **Smoking** | **Alcohol** | **Caffeine** |
| Extraversion | 1.74 (1.16-2.60), p=0.007 | 2.14 (1.44-3.17), p<0.001 | 0.98 (0.68-1.41), p=0.91 | 1.87 (1.21-2.88), p=0.005 | 1.72 (1.13-2.61), p=0.01 | 0.94 (0.63-1.39), p=0.76 |
| Neuroticism | 1.30 (0.94-1.80), p=0.11 | 0.55 (0.40-0.76), p<0.001 | 1.27 (0.94-1.72), p=0.12 | 1.21 (0.84-1.75), p=0.30 | 0.70 (0.49-1.00), p=0.05 | 1.31 (0.93-1.84), p=0.12 |
| Agreeableness | 0.23 (0.13-0.56), p<0.001 | 1.11 (0.53-2.34), p=0.78 | 0.89 (0.44-1.80), p=0.74 | 0.26 (0.12-0.57), p=0.001 | 0.84 (0.38-1.84), p=0.66 | 0.95 (0.45-1.98), p=0.88 |
| Openness | 1.16 (0.68-2.00), p=0.58 | 2.98 (1.75-5.06), p<0.001 | 0.87 (0.53-1.43), p=0.57 | 1.28 (0.73-2.26), p=0.39 | 2.19 (1.26-3.80), p=0.005 | 0.84 (0.50-1.42), p=0.52 |
| Conscientiousness | 0.61 (0.33-1.15), p=0.13 | 2.04 (1.09-3.81), p=0.03 | 1.88 (1.04-3.41), p=0.04 | 0.61 (0.31-1.19), p=0.15 | 1.35 (0.69-2.64), p=0.38 | 2.20 (1.16-4.17), p=0.02 |

The number of pack years smoked, current weekly alcohol intake and current daily caffeine intake were used for analysis.

To determine the effect of each aspect of personality on each of these behaviours, all the subjects regardless of disease status were pooled together into the model. The total score for each personality trait was divided into quintiles with ordinal logistic regression then used to calculate odds ratios. The co-variates for each regression model are listed in the table.

# Supplemental Figure 1

This figure shows the total unadjusted score for each of the five personality domains, split by groups based on case status.
